# Software review

**DOI:** 10.1155/S1463924600000365

**Published:** 2000


Journal of Automated Methods & Management in Chemistry, Vol. 22, No. 6 (November–December 2000) p. 203

### Software review

### New Software for Workstation and Data Security

Zymark Corporation announces the availability of new Version 2.0 software for the Tablet Processing Workstation, TPW™ II. The software, available for shipment with new instruments or as an update, provides enhanced TPW Controller workstation security along with data security that is compliant with 21 CFR Part 11 regulations.

Developed to function within the Windows® NT Version 4.0 operating system, the TPW software Version 2.0 is written as a 32-bit application and utilizes Windows NT security manager functions that allow the NT administrator to define multiple levels of security and system access through password control. The software includes a SQL: Server 7.0 Relational Database that is used for all raw data and event logging. It also provides secure and uneditable raw data storage and maintains data records consistent with 21 CFR Part 11. Raw data and events can be queried and custom reports generated.

The new TPW software includes enhancements to the TPW core application. A dispersion module cycling routine allows the user to program an up and down movement of the vessel during the extraction cycle, permitting easier extraction of sticky, or difficult-to-disrupt dosage forms. The software will also serve as the basis for several optional add-in products, either already released or under further development for future release. These add-ins include a direct interface for the TPW II to the Hewlett-Packard ChemStation, an Advanced Data Channels Organizer that permits the re-direction of selected TPW II data to any network device in ASCII, EXCEL or ODBC formats, and a direct interface of the TPW II to the Waters Millennium Chromatography Data Manager.

Zymark impacts the world by advancing the ability to discover, develop and market safe and effective drugs that lengthen and improve the quality of life for everyone. Zymark's employees are active in 33 countries and their combined expertise in Pharmaceutical Analysis and Laboratory Automation makes Zymark the world supplier in this application area.

*For more information please contact Lynda Thomas, Marketing Communications. Tel: +1 (508) 497-6549; e-mail: lynda.thomas@zymark.com.*

